# Numerical indices based on circulating tumor DNA for the evaluation of therapeutic response and disease progression in lung cancer patients

**DOI:** 10.1038/srep29093

**Published:** 2016-07-06

**Authors:** Kikuya Kato, Junji Uchida, Yoji Kukita, Toru Kumagai, Kazumi Nishino, Takako Inoue, Madoka Kimura, Shigeyuki Oba, Fumio Imamura

**Affiliations:** 1 Department of Molecular and Medical Genetics, Research Institute, Osaka Medical Center for Cancer and Cardiovascular Diseases, Osaka, Japan; 2 Department of Thoracic Oncology, Osaka Medical Center for Cancer and Cardiovascular Diseases, Osaka, Japan; 3 Graduate School of Informatics, Kyoto University, Kyoto, Japan

## Abstract

Monitoring of disease/therapeutic conditions is an important application of circulating tumor DNA (ctDNA). We devised numerical indices, based on ctDNA dynamics, for therapeutic response and disease progression. 52 lung cancer patients subjected to the EGFR-TKI treatment were prospectively collected, and ctDNA levels represented by the activating and T790M mutations were measured using deep sequencing. Typically, ctDNA levels decreased sharply upon initiation of EGFR-TKI, however this did not occur in progressive disease (PD) cases. All 3 PD cases at initiation of EGFR-TKI were separated from other 27 cases in a two-dimensional space generated by the ratio of the ctDNA levels before and after therapy initiation (mutation allele ratio in therapy, MART) and the average ctDNA level. For responses to various agents after disease progression, PD/stable disease cases were separated from partial response cases using MART (accuracy, 94.7%; 95% CI, 73.5–100). For disease progression, the initiation of ctDNA elevation (initial positive point) was compared with the onset of objective disease progression. In 11 out of 28 eligible patients, both occurred within ±100 day range, suggesting a detection of the same change in disease condition. Our numerical indices have potential applicability in clinical practice, pending confirmation with designed prospective studies.

Circulating tumor DNA (ctDNA) is the cell-free DNA released from dying cancer cells^1^, and represents an emerging field of cancer research. Because ctDNA appears more frequently in advanced cancers than in early cancers^2^ and its level is generally considered to correlate with the tumor burden, monitoring of disease/therapeutic conditions is regarded as the foremost application of ctDNA^3^. Usually, cancer-related mutations are identified in primary lesions, and these serve as markers to detect ctDNA. Therapy-resistant mutations of target genes were identified with several agents, and these may be used to monitor acquired resistance^4^,^5^,^6^,^7^,^8^.

In the case of advanced non-small cell lung cancer, ctDNA has been extensively explored for genotyping of *EGFR*. Due to a strong correlation between *EGFR* activating mutations and the efficacy of EGFR tyrosine kinase inhibitors (EGFR-TKIs)^9^,^10^, the detection of mutations is indispensable for therapeutic decision making. Introduction of the next-generation EGFR-TKIs^11^,^12^ targeting EGFR with the T790M^13^ resistant mutation necessitates the genotyping of the T790M locus. ctDNA containing EGFR-activating mutations and that containing the T790M mutation can serve as a metric for all cancer cells and therapy-resistant cells, respectively. ctDNA analysis in the EGFR-TKI treatment is advantageous over that in treatments using the other agents, which requires the identification of marker mutations to follow the whole amount of ctDNA.

Observation of ctDNA dynamics is often subjective. To enable the objective evaluation of ctDNA dynamics, it is desirable to have simple numerical indices that summarize information of individual events. Such indices could be directly used in clinical practice once their utility is established. In the case of advanced cancer, evaluation of therapeutic responses and disease progression is important from a clinical viewpoint. In addition, the comparison should be performed with data obtained from an unbiased patient population, simulating real clinical practice.

We constructed a detection system for *EGFR* mutations in ctDNA using deep sequencing with a massively parallel DNA sequencer^5^. This system is among the most intensively validated assay systems for ctDNA^14^. Using this system, we conducted a prospective exploratory study to follow temporal changes of ctDNA levels under a real clinical setting. The general features of the data were previously described^15^. In this report, we propose two numerical indices to extract relevant information from ctDNA dynamics for clinically important events. These are a numerical index for the evaluation of the therapeutic response, and an index to estimate the onset of disease progression. The performance of these indices was evaluated using the current standards, namely the Response Evaluation Criteria In Solid Tumors (RECIST)^16^. We demonstrate that these indices, particularly the therapeutic response index, appear to be useful for studying ctDNA dynamics and should be further investigated with designed prospective studies.

## Results

### Patient and sample populations

In total, 52 patients participated in the study. The clinical characteristics of this patient population are shown in Table 1. Patient information corresponds to the initiation of the EGFR-TKI treatment. The total number of blood samples was 530. The initial PCR amplification of *EGFR* exon fragments was successful in all the samples, and mutation data were obtained from all the samples.

### Evaluation of the initial response to EGFR-TKI

We examined whether ctDNA dynamics could be used to evaluate the effect of EGFR-TKI treatment. As 5 patients lacked samples of the corresponding time points, 47 patients were eligible for the analysis. Thirty patients (63.8%), whose initial levels of ctDNA exceeded the threshold, were forwarded to the following quantitative analysis. Clinical characteristics of this patient subpopulation are shown in Table 1.

**Figure 1:**
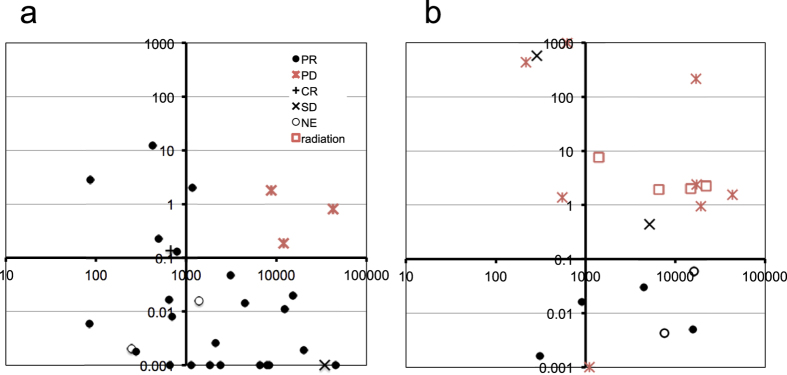
Quantitative changes of ctDNA levels during therapeutic changes. Vertical axis, mutation allele ratio in therapy (MART). Horizontal axis, average of PM scores before and after initiation of therapies. (**a**) Response to the initial EGFR-TKI treatment. (**b**) Response to various therapies after the first disease progression. MART > 1000 and <0.001 are converted to 1000 and 0.001, respectively.

Mutation allele ratio in therapy (MART) is defined as the ratio of the PM score of the activating mutation after the initiation of therapy to that before the therapy, and is used as an index for therapeutic response. In the most cases, the initial high ctDNA levels decreased rapidly during the 2 weeks after the initiation of EGFR-TKI, but some cases continued to decrease until 4 weeks. For the calculation of MART, PM scores of 2 weeks and 4 weeks were used for 23 and 7 cases, respectively. MART is plotted in Supplementary Figure S1a, grouping the patients into PD and PR/complete response (CR)/SD/not evaluable (NE) cases. MART of all three PD cases exceeded 0.1. On the contrary, MART exceeded 0.1 in only 6 out of 27 PR/CR/SD/NE cases. PM scores of these 9 cases are plotted in Supplementary Figure 1b. PM scores of PD cases were significantly higher than those of PR/CR cases. In the case of low ctDNA levels, the number of molecules in blood samples may fluctuate among sampling events, leading to fluctuations in PM scores. We therefore used the average of PM scores of the two assays as a second parameter to assess the reliability of MART. In a two-dimensional space generated by MART and PM scores, PD cases segregated in the upper right part of the plot (Fig. 1a).

### Identification of cases eligible for long-term temporal analysis

Next, we examined whether ctDNA dynamics could be used to estimate the onset of disease progression during the EGFR-TKI treatment. The target period was from 1 month after the initiation of EGFR-TKI to the time of the detection of disease progression. Only patients who developed objective disease progression (PD) during the EGFR-TKI treatment were eligible for this analysis. 11 patients stopped the EGFR-TKI treatment before PD due to PD during the initial response (3 patients), death (3 patients), adverse effects (4 patients) and patient intension (1 patient). Six patients did not develop PD during the observation time (median, 358 days; minimum, 198 days; maximum, 747 days). The remaining 35 patients developed PD. Statistics of the time to develop PD in these cases is as follows: median, 335 days; minimum, 57 days; maximum, 838 days.

To exclude cases with insufficient data, we chose cases that had 4 or more samples within a span of 300 days that included the PD time point. Twenty-eight cases met this criterion. Their clinical information is provided in Table 2 and summary statistics are in Table 1 (n = 351; sampling time interval: median = 43 days, lower quartile = 22 days, upper quartile = 63 days).

### Correlation between ctDNA dynamics and the onset of disease progression

**Figure 2:**
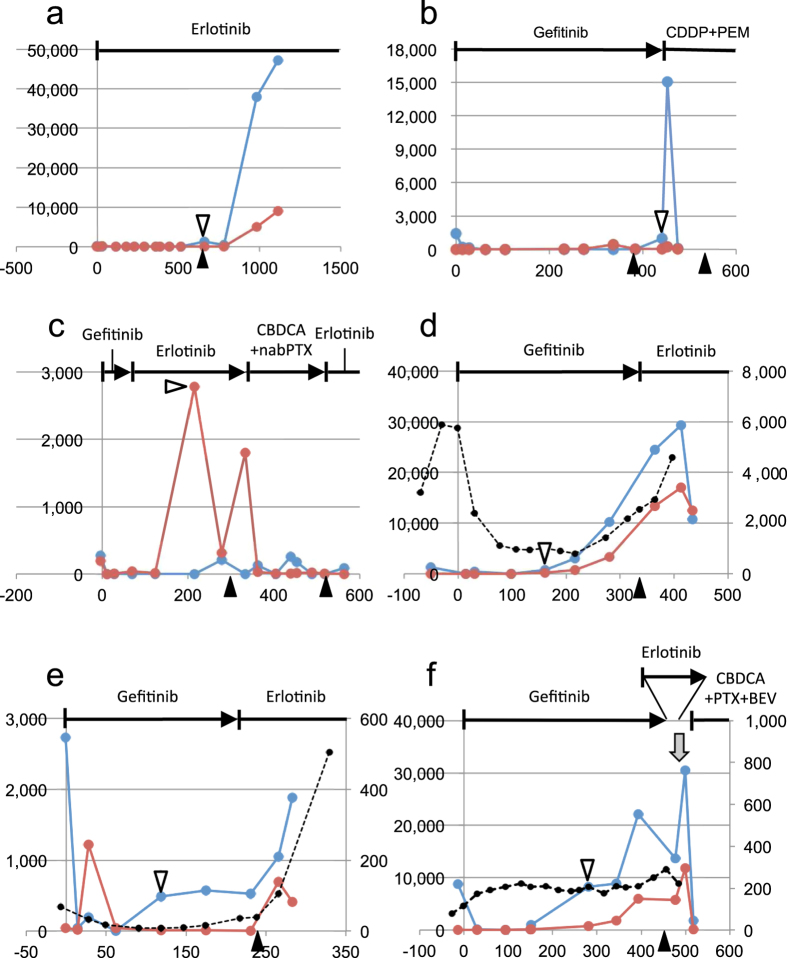
ctDNA dynamics of type I and II patients. Vertical axis, PM score for *EGFR* mutations (left) or protein concentration (μg/mL) for CEA (right). Horizontal axis, days from initiation of EGFR-TKI. Horizontal lines at the top of each panel indicate treatment, vertical bars indicate initiation of therapy, and arrowheads indicate termination of therapy. Gray arrows below the horizontal lines indicate radiotherapy. Black arrowheads in the bottom of each panel indicate PD points. White arrowheads in each panel indicate IP points. Blue lines indicate activating mutations (exon 19 deletion or L858R). Red lines indicate T790M. Black broken lines indicate CEA. For patients who had no data exceeding LOD (PM score) or whose data were within the normal range (CEA), data were not presented in graphs. (**a**) Patient 1 (type I). (**b**) Patient 5 (type I). (**c**) Patient 11 (type I). (**d**) Patient 12 (type II). (**e**) Patient 13 (type II). (**f**) Patient 14 (type II). Abbreviations are as follows: CDDP, cisplatin; PEM, pemetrexed; CBDCA, carboplatin; nabPTX, nab-paclitaxel; PTX, paclitaxel; BEV, bevacizumab.

**Figure 3:**
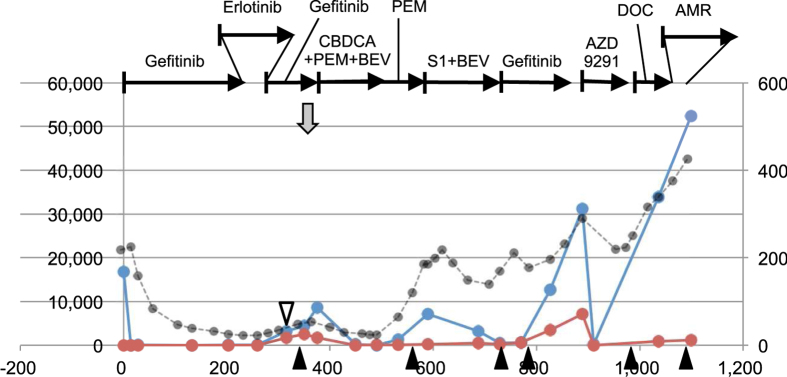
ctDNA dynamics of patient 9 (type I) with long observation time after the first disease progression. The details of panels are the same as those in Fig. 2. Abbreviations are as follows: DOC, docetaxel; AMR, amurubicin.

Our previous study with a retrospective data set revealed that ctDNA levels of activating and T790M mutations were suppressed during the EGFR-TKI treatment until the onset of objective disease progression, but increased thereafter^17^. To compare ctDNA dynamics with objective disease progression identified by medical imaging, we defined the initial positive (IP) point as the initial time point when ctDNA levels starts to exceed the threshold after the suppression. We identified the IP point for each patient and compared it with the time point of objective disease progression (PD point). Schematic representation of ctDNA dynamics is presented in Supplementary Figure S2 to facilitate the understanding of IP and PD points. IP and PD points were shown as time intervals from the date of the initiation of EGFR-TKI, represented by days (Table 2). In patients 1–11, named “type I” patients, the time interval between IP and PD points was within ±100 day range (IP from PD, Table 2), and there was a single data point or were no data points in the interval (Figs 2a–c and 3, Supplementary Figure S3a–g). Considering time intervals between diagnostic tests, ctDNA dynamics and medical imaging were likely to capture the same change in disease condition. In 6 patients, the ctDNA level decreased upon initiation of new therapies (Figs 2b,c and 3, Supplementary Figure S3d,e,g).

In patients 12–15, named “type II” patients, the IP point preceded the PD point in time by more than 100 days (IP from PD, Table 2), and there were two data points in the interval (Fig. 2d–f, Supplementary Figure S3h). In these patients, ctDNA dynamics diverged from medical imaging and elevated ahead of objective disease progression. In contrast to the uniformity of the ctDNA dynamics in type I patients, those in type II patients were more variable. In patient 12, the ctDNA levels constantly increased after the IP point (Fig. 2d). In patient 13, the ctDNA levels of the activating mutation elevated and maintained a certain level until the PD point, and then increased, with an accompanying increase in the T790M ctDNA (Fig. 2e). In patient 15, the pattern was similar to that of patient 13, but the ctDNA did not increase upon PD probably due to the intervention of cytotoxic agents (Supplementary Figure S3h). The temporal pattern of patient 14 was complicated partly due to the intervention of other therapies (Fig. 2f).

Both the activating and the T790M mutations were elevated after the IP point in 8 patients (Figs 2a,b,d–f and 3, Supplementary Figure S3a,c,d). In these patients, the ctDNA level of T790M was constantly lower than that of the activating mutation, but often elevated later. We noted the solitary appearance of the T790M ctDNA in patient 11 (Fig. 2c). In this case, the IP point was determined with the T790M ctDNA. We previously observed this type of cancer cell subpopulations^17^, which is not likely to be rare.

Patients 16–28 had no elevation related to PD, and were classified as “type III” (Table 2). Representative examples of the ctDNA dynamics are shown in Supplementary Figure 4. On the whole, the numbers of type I, type II, and type III patients were 11 (39.3%), 4 (14.3%) and 13 (46.4%), respectively. Solitary peak of activating mutations appeared in patients 2 and 28 (Supplementary Figures 3a and 4c; Table 2). Although the peak could be regarded as a false positive of the IP point, its incidence was low, and therefore, it was not likely to evoke confusion in the identification of IP points.

We measured the level of carcinoembryonic antigen (CEA) in all cases. Increases of CEA in parallel with objective disease progression were observed in 9 out of the 28 patients (32.1%).

### Response to various therapies

Disease progression is often accompanied by therapy changes. Patient 9 had a long history of different therapies after the first disease progression, and is presented as an example (Fig. 3). This patient was treated with various agents including re-challenge of gefitinib. Both the activating mutation and the T790M ctDNA was elevated after the first PD, but these were soon suppressed with cytotoxic agents. During the treatment with various cytotoxic agents, the T790M ctDNA was suppressed, but the activating mutation was elevated in parallel with disease progression. Upon gefitinib, the T790M ctDNA increased, but AZD9291, EGFR-TKI targeted to T790M, suppressed the T790M. The ctDNA dynamics indicated that the T790M ctDNA represented a cancer cell population with drug susceptibility distinct from that of the major cancer cell population. Other patients with a long history of disease progression, that is patients 10 and 15, are shown in Supplementary Figures S3g,h.

All therapeutic changes after the first disease progression are presented in Table 3. These events were classified according to objective response, except for radiation therapy, and plotted in the two-dimensional space generated by MART and the average of PM scores (Fig. 1b). For the calculation, we used the time points adjacent to the initiation of a therapy. Relevant data are presented in Table 3. PD and SD cases segregated in the upper part of the plot, and PR cases segregated in the lower part with a single exception. Applying the threshold (MART = 0.1) deduced in the analysis of EGFR-TKI, the accuracy to discriminate between PD/SD and CR/PR was 94.7% (95% confidence interval, 73.5–100). Collecting the results of EGFR-TKI and these therapies, MART is the primary parameter to evaluate therapeutic response. The merit of the second parameter, that is, the average of the ctDNA levels of the two assays, was not clear in this data set, and may be dispensable. Unlike anti-cancer agents, radiation therapy did not change ctDNA dynamics, and segregated in the upper right part of the two-dimensional space.

## Discussion

The main purpose of the numerical indices is to offer objective methods to correlate ctDNA dynamics with clinical status; that is, therapeutic response and onset of objective disease progression. So far, the analysis of temporal changes in the ctDNA dynamics is simple observation of temporal patterns. We demonstrated here that subjective observation could be replaced with simple numerical indices. In particular, MART agreed well with the therapeutic response evaluated with the RECIST criteria. The results of this study are promising and may proceed to further confirmatory studies.

For the evaluation of the therapeutic response, MART served as the index and exhibited a good correlation with medical imaging. The second analysis on the various therapies may be regarded as a validation of the classifier constructed using the results of the first analysis, demonstrating good predictability. The response of ctDNA is rapid because of its extremely short half-life^18^,^19^. Therefore, its main advantage over medical imaging is the early access to information. For example, in the case of initial EGFR-TKI, the ctDNA data for most cases was obtained 2 weeks after therapy initiation.

For the onset of disease progression, we devised an IP point as the index. In type I patients, the IP point was likely to indicate the same change in the disease condition as did the objective disease progression. In type II patients, the IP point preceded the onset of the objective disease progression. It was not clear whether the early elevation of the ctDNA level in type II patients was a sign of disease progression or a consequence of other changes in the disease condition. Although ctDNA dynamics may represent the disease condition more precisely, the presence of type II patients introduces some uncertainty to the predictability of the IP index. We note that the current border between types I and II is temporary, and we need to examine this issue in further detail.

Importantly, the ctDNA level of the activating mutations is more informative than that of T790M, and it uses both the MART and the IP point in most cases. The T790M ctDNA, which represents a part of the cancer cell population, is useful for identifying subpopulations with unique characteristics, but ctDNA levels of activating mutations are indispensable for the entire view of the ctDNA dynamics.

From a number of studies comparing genotyping of biopsies and peripheral blood^14^,^20^,^21^, the detection rate of ctDNA in advanced lung cancer is 60–70%. Our detection rate before the initial EGFR-TKI treatment was within this range. Our rate in the long-term temporal analysis was 53.6%, but this figure may be an underestimation, because approximately half of type III patients had a short observation time after the PD point. The presence of patients without detectable ctDNA would be the main disadvantage to reap clinical benefits. In contrast, the simplicity of the MART and the IP point would be an advantage over medical imaging, which often provides complicated information.

To apply this approach to other cancers, it is necessary to develop new technologies to detect multiple mutations without screening of mutations in the primary lesions. We expect that recent advances in genomic technologies^22^ will solve this problem soon.

## Methods

### Patients

Lung cancer patients with *EGFR* activating mutations subjected to the first EGFR-TKI treatment regardless of any prior treatments were recruited for this study (University Hospital Medical Information Network Clinical Trials Registry UMIN000006764) from November 2011 to March 2014 in the Osaka Medical Center for Cancer and Cardiovascular Diseases. Written informed consent was obtained from all participants. This study was approved by the ethics committee of the Osaka Medical Center for Cancer and Cardiovascular Diseases. The methods were carried out in accordance with the approved guidelines.

### Blood sampling

Blood sampling for the ctDNA assay was scheduled to be performed before the initiation of EGFR-TKI treatment, as well as two and four weeks (14 and 28 days) after the initiation. The timing allowed a leeway of ±4 days, not counting holidays. Sampling after this period was intended each 2 months and was continued at least until the onset of objective disease progression and beyond this point when possible. Blood sampling was terminated on June 30 2014.

### Clinical evaluation of therapeutic response and disease progression

Evaluation of response to therapy was done using RECIST version 1.1, and in the case of initial EGFR-TKI, approximately two months after treatment initiation. Evaluation of disease progression during the EGFR-TKI treatment was also based on the RECIST criteria. Additionally, criteria recommended in the Guidelines for Treatment and Diagnosis of Lung Cancer (the Japanese Lung Cancer Society) were applied.

### ctDNA assay

The assay system searches mutations by deep sequencing, that is, sequencing a large number of gene fragments. Exons 19, 20 and 21 of the *EGFR* gene were independently amplified with PCR from patient plasma DNA, and deep sequencing was performed with the Ion Torrent PGM (Thermo Fisher Scientific, Waltham, MA, USA)^23^. A diagnostic score, termed the plasma mutation (PM) score, was defined as the number of reads with deletions (exon 19 deletions) or substitutions (exon 20, T790M; exon 21; L858R, L861Q) in 100,000 reads. We deduced parameters corresponding to the limit of detection (LOD) and limit of quantification (LOQ)^5^, and used them to define a threshold for mutation detection. According to the results of a previous study^14^, the threshold for the initial level, that is, before the initiation of EGFR-TKI, was set as LOQ for exon 19 deletion and LOD for L858R. For the analysis of disease progression, we chose a conservative approach to set the threshold of detection as LOQ (PM score = 300). The multi-institute study^14^ conducted along with this study indicated that the possibility of false positive was negligible under this setting: the estimates of false-positive rates for exon 19 deletion, L858R, and T790M were 2%, 0%, and 1%, respectively.

The laboratory procedures of the ctDNA assay were the same as previously described^5^ except that the current study used the latest versions of sequencing reagents. The assay was performed in the order of the sampling date from November 2012 to September 2014 at a rate of 12–24 samples per week.

## Additional Information

**How to cite this article**: Kato, K. *et al*. Numerical indices based on circulating tumor DNA for the evaluation of therapeutic response and disease progression in lung cancer patients. *Sci. Rep*. **6**, 29093; doi: 10.1038/srep29093 (2016).

## Supplementary Material

Supplementary Information

## Figures and Tables

**Table 1:** Patient characteristics.

	All patients	Initial response	Disease progression
Total number of cases	52	30	28
Sex			
Male	15	8	9
Female	37	22	19
Age			
<49	7	4	7
50–59	8	5	4
60–69	18	12	8
70–79	17	7	8
80–89	2	2	1
Stage			
IIIA	4	3	3
IIIB	7	5	2
IV	41	22	23
Mutation in biopsy sample			
exon 19 deletion	26	15	16
L858R	23	14	11
double	1	0	0
L861Q	1	1	1
G719C	1	0	0
Treatment before EGFR-TKI			
none	25	15	15
surgery	3	1	3
surgery/radiation	6	4	2
surgery/chemotherapy	3	1	3
radiation	7	5	2
radiation/chemotherapy	4	1	2
chemotherapy	4	3	1
EGFR-TKI			
gefitinib	41	22	24
erlotinib	11	8	4
Effect of EGFR-TKI			
complete response (CR)	2	1	1
partial response (PR)	36	23	22
stable disease (SD)	7	1	2
progressive disease (PD)	3	3	0
not evaluable (NE)	4	2	3

**Table 2:** Clinical information of the patients subjected to temporal analysis and summary statistics of disease progression.

Patient ID	Figure ID	Type	Stage	Sage	Sex	Activating mutation	Initial response to EGFR-TKI	Prior treatment	ctDNA before EGFR-TKI	Last data point (1)	PD point (1)	IP point (1)	IP from PD (2)	Solitary peak (1)
1	2A	type I	IIIA	77	F	L858R	PR	surgery/chemotherapy	+	1115	659	659	0	−
2	S3A	type I	IV	39	F	exon 19 deletion	PR	curative radiation/chemotherapy	+	437	372	308	−64	112 (exon 19 deletion)
3	S3B	type I	IV	80	M	L858R	PR	none	+	440	296	347	51	−
4	S3C	type I	IV	62	M	exon 19 deletion	PR	none	+	228	180	129	−51	−
5	2B	type I	IV	69	F	L858R	PR	none	+	475	383	441	58	336 (T790M)
6	S3D	type I	IV	63	M	exon 19 deletion	PR	curative surgery	+	659	303	385	82	−
7	S3E	type I	IV	68	F	exon 19 deletion	PR	none	+	229	172	171	−1	−
8	S3F	type I	IIIB	39	F	exon 19 deletion	SD	curative surgery	−	718	159	252	93	−
9	3	type I	IV	37	M	exon 19 deletion	PR	none	+	1099	339	315	−24	−
10	S3G	type I	IV	73	F	L858R	PR	local radiation	+	865	325	360	35	143 (T790M)
11	2C	type I	IV	78	F	exon 19 deletion	SD	none	−	563	299	215	−84	−
12	2D	type II	IV	60	F	exon 19 deletion	PR	chemotherapy	+	434	336	161	−175	−
13	2E	type II	IIIA	66	F	L858R	NE	none	+	283	238	119	−119	−
14	2F	type II	IV	59	M	exon 19 deletion	PR	none	+	518	456	152	−304	−
15	S3H	type II	IIIB	56	F	L861Q	PR	none	+	761	335	102	−233	−
16	−	type III	IIIA	53	F	exon 19 deletion	PR	surgery/chemotherapy	−	700	558	n/a	n/a	−
17	−	type III	IV	73	F	L858R	NE	surgery/local radiation	+	75	57	n/a	n/a	−
18	−	type III	IV	61	F	exon 19 deletion	PR	none	−	489	375	n/a	n/a	−
19	−	type III	IV	49	M	L858R	PR	local radiation	+	560	202	n/a	n/a	−
20	−	type III	IV	49	M	exon 19 deletion	CR	surgery	−	874	838	n/a	n/a	−
21	−	type III	IV	72	M	L858R	PR	surgery radiataion	+	796	421	n/a	n/a	−
22	S4A	type III	IV	40	F	exon 19 deletion	PR	none	+	780	530	n/a	n/a	−
23	S4B	type III	IV	62	F	exon 19 deletion	PR	curative radiation/chemotherapy	−	667	350	n/a	n/a	−
24	−	type III	IV	74	M	L858R	PR	none	n/a	215	196	n/a	n/a	−
25	−	type III	IV	41	F	L858R	NE	curative surgery/chmotherapy	n/a	1075	591	n/a	n/a	−
26	−	type III	IV	73	F	exon 19 deletion	PR	none	−	554	290	n/a	n/a	−
27	−	type III	IV	54	F	L858R	PR	none	+	303	290	n/a	n/a	−
28	S4C	type III	IV	71	F	exon 19 deletion	PR	none	+	334	334	n/a	n/a	110 (exon 19 deletion/T790M)

(1) Days counted from initiation of EGFR-TKI. (2) Days counted from PD point to IP point. n/a, data not available or not applicable.

**Table 3:** Response to various therapies after the first disease progression.

Patient ID	Figure ID	Type	Marker mutation	Treatment	Initiation of new treatment (1)	Assay before new treatment (2)	Assay after new treatment (2)	Response evaluation (2)	PM score of the first assay	PM score of the second assay	MART	Objective response
5	2B	type I	L858R	CDDP+PEM	454	−1	21	28	15057	65	0.004	NE
6	S3D	type I	exon 19 deletion	local radiation (brain)	527	−20	68	n/a	9865	19738	2.000	radiation
6	S3D	type I	exon 19 deletion	CBDCA+nabPTX	644	−49	15	42	19738	18724	0.949	PD
7	S3E	type I	exon 19 deletion	CDDP+PEM	172	−1	57	57	2206	n.d.	0.000	PD
8	S3F	type I	exon 19 deletion	CDDP+PEM	209	−5	43	93	n.d.	572	572	SD
9	3	type I	exon 19 deletion	local radiation (brain)	358	−8	17	n/a	4461	8619	1.932	radiation
9	3	type I	exon 19 deletion	CBDCA+PEM+BEV	378	−3	71	84	8619	261	0.030	PR
9	3	type I	exon 19 deletion	PEM	504	−14	28	56	n.d.	1254	1254	PD
9	3	type I	exon 19 deletion	S1+BEV	589	0	98	49	7171	3153	0.440	SD
9	3	type I	exon 19 deletion	gefitinib	729	0	41	56	464	639	1.377	PD
9	3	type I	exon 19 deletion	AZD9291	889	0	21	36	31119	157	0.005	PR
9	3	type I	exon 19 deletion	docetaxel	994	−84	42	21	157	33872	216	PD
9	3	type I	exon 19 deletion	AMR	1064	−28	35	21	33872	52361	1.546	PD
10	S3G	type I	L858R	gefitinib+GEM	353	−28	7	10	n.d.	431	431	PD
10	S3G	type I	L858R	gefitinib+PEM	381	−7	42	71	n.d.	n.d.	n/a	NE
10	S3G	type I	L858R	local radiation (illium)	531	−45	19	n/a	326	2484	7.620	radiation
11	2C	type I	T790M	CBDCA+nabPTX	334	−1	28	133	1797	29	0.016	PR
11	2C	type I	T790M	erlotinib	518	−30	45	47	n.d.	n.d.	n/a	SD
12	2D	type II	exon 19 deletion	erlotinib	336	−56	28	61	10176	24447	2.402	PD
14	2D	type II	exon 19 deletion	local radiation (bone)	495	−18	4	n/a	13650	30448	2.231	radiation
14	2F	type II	exon 19 deletion	CBDCA+PTX+BEV	510	−11	8	n/a	30448	1789	0.059	NE
15	S3H	type II	L861Q	CDDP+PEM	363	−2	32	76	620	n.d.	0.002	PR
15	S3H	type II	L861Q	PEM	489	−94	42	30	n.d.	n.d.	n/a	SD

(1) Days counted form initiation of EGFR-TKI. (2) Days counted form initiation of new treatment. n/a, not applicable; n.d., not detected.

MART is calculated assgining 1 for"n.d." of single "n.d." cases. GEM, gemcitabine.
