# Supplementary material for: Numerical indices based on circulating tumor DNA for the evaluation of therapeutic response and disease progression in lung cancer patients
Source: Sci Rep. 2016 Jul 6;6:29093. doi: 10.1038/srep29093 (PMC4933907; doi:10.1038/srep29093)
Supplement: Supplementary Information [file srep29093-s1.pdf]

Supplementary Information

Numerical indices based on circulating tumor DNA dynamics for the evaluation of therapeutic response and disease progression in lung cancer patients

Kikuya Kato[1], Junji Uchida[2], Yoji Kukita[1], Toru Kumagai[2], Kazumi Nishino[2], Takako Inoue[2], Madoka Kimura[2], Shigeyuki Oba[3] and Fumio Imamura[2]

[1]Department of Molecular and Medical Genetics, Research Institute, Osaka Medical Center for Cancer and Cardiovascular Diseases, Osaka, Japan.

[2]Department of Thoracic Oncology, Osaka Medical Center for Cancer and Cardiovascular Diseases, Osaka, Japan.

[3]Graduate School of Informatics, Kyoto University, Kyoto, Japan.

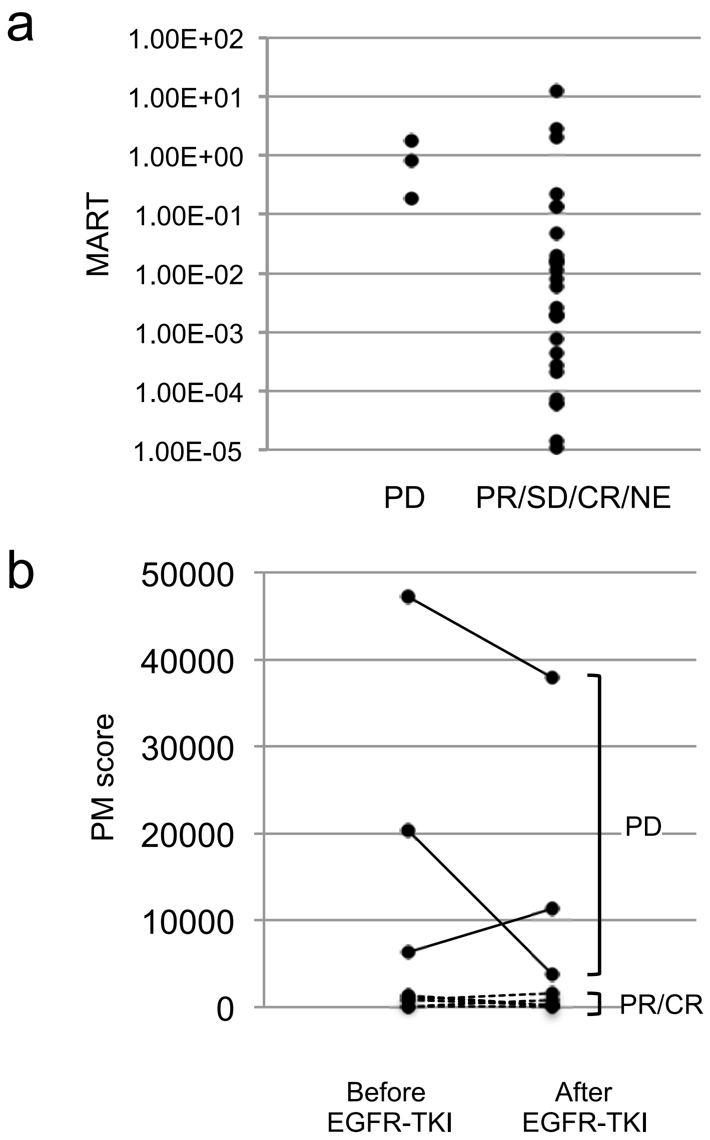

Supplementary Figure S1. Changes of ctDNA levels due to initiation of EGFR-TKI. A, Mutation allele ratio in therapy (MART). B, PM scores before and after the initiation of EGFR-TKI.

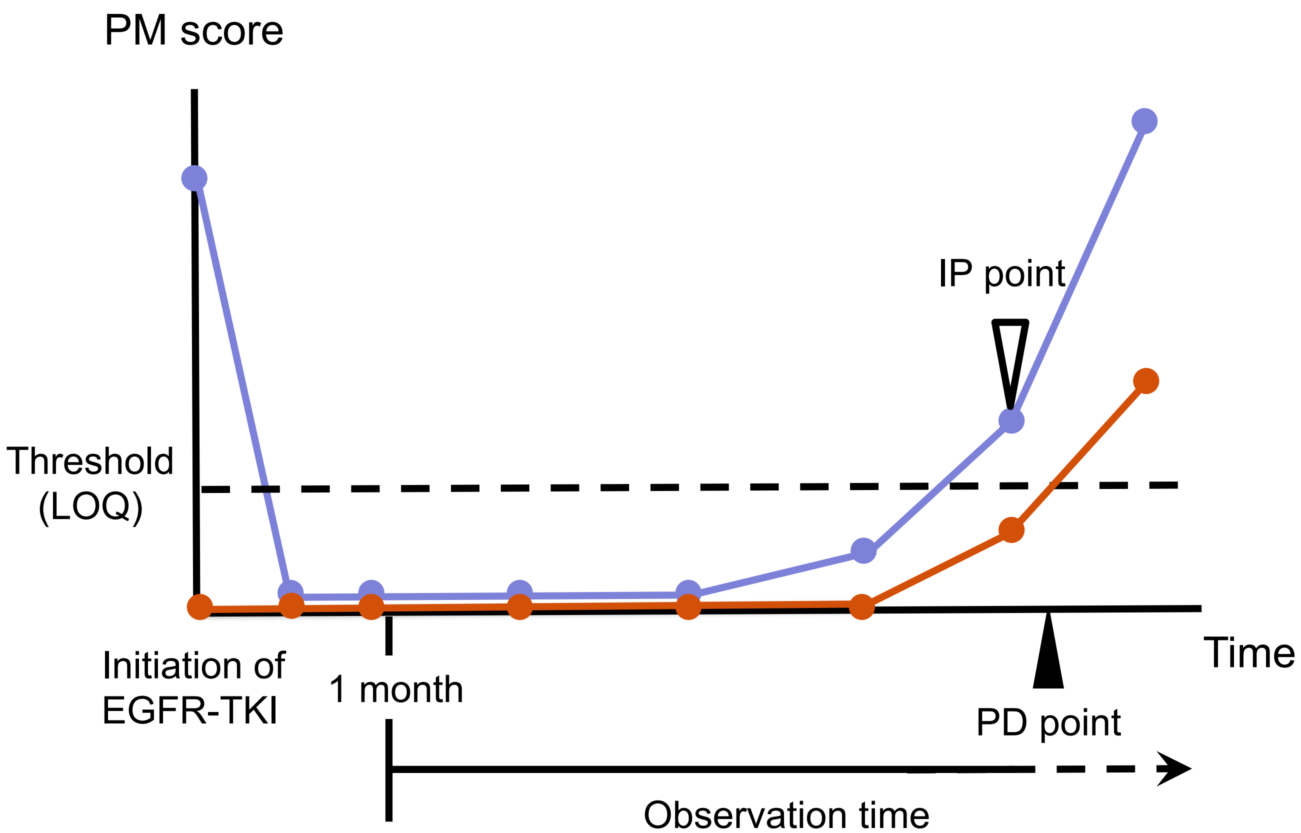

Supplementary Figure S2. Schematic representation of circulating tumor DNA dynamics in the EGFR-TKI treatment. Blue line, activating mutation; red line, resistant mutation.

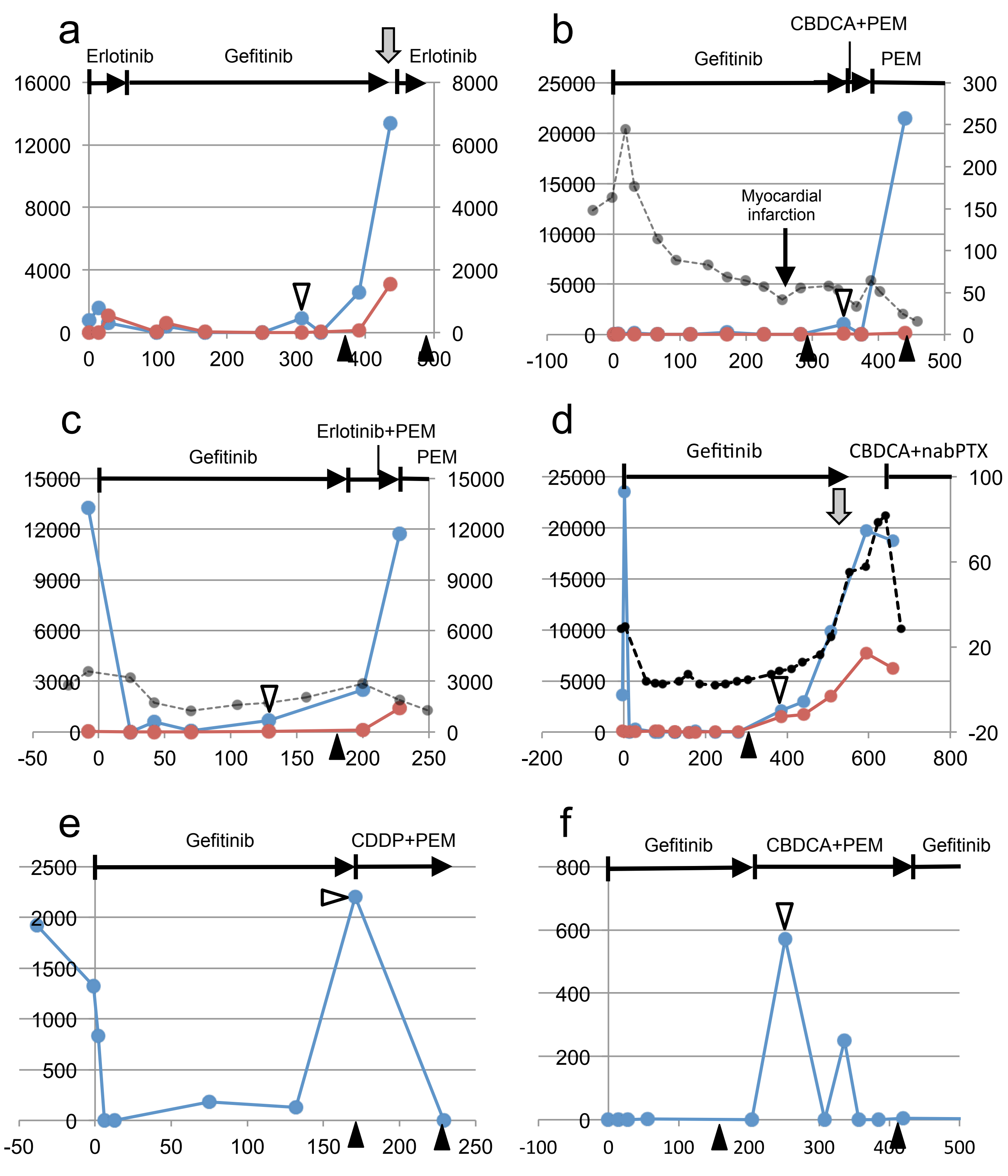
a
Erlotinib
Gefitinib
Erlotinib
16000
12000
8000
4000
0
8000
6000
4000
2000
0
0
100
200
300
400
500
b
CBDCA+PEM
Gefitinib
PEM
25000
20000
15000
10000
5000
0
300
250
200
150
100
50
0
Myocardial
infarction
-100
0
100
200
300
400
500
c
Erlotinib+PEM
Gefitinib
PEM
15000
12000
9000
6000
3000
0
-50
0
50
100
150
200
250
d
Gefitinib
CBDCA+nabPTX
25000
20000
15000
10000
5000
0
100
60
20
-20
-200
0
200
400
600
800
e
Gefitinib
CDDP+PEM
2500
2000
1500
1000
500
0
-50
0
50
100
150
200
250
f
Gefitinib
CBDCA+PEM
Gefitinib
800
600
400
200
0
-100
0
100
200
300
400
500

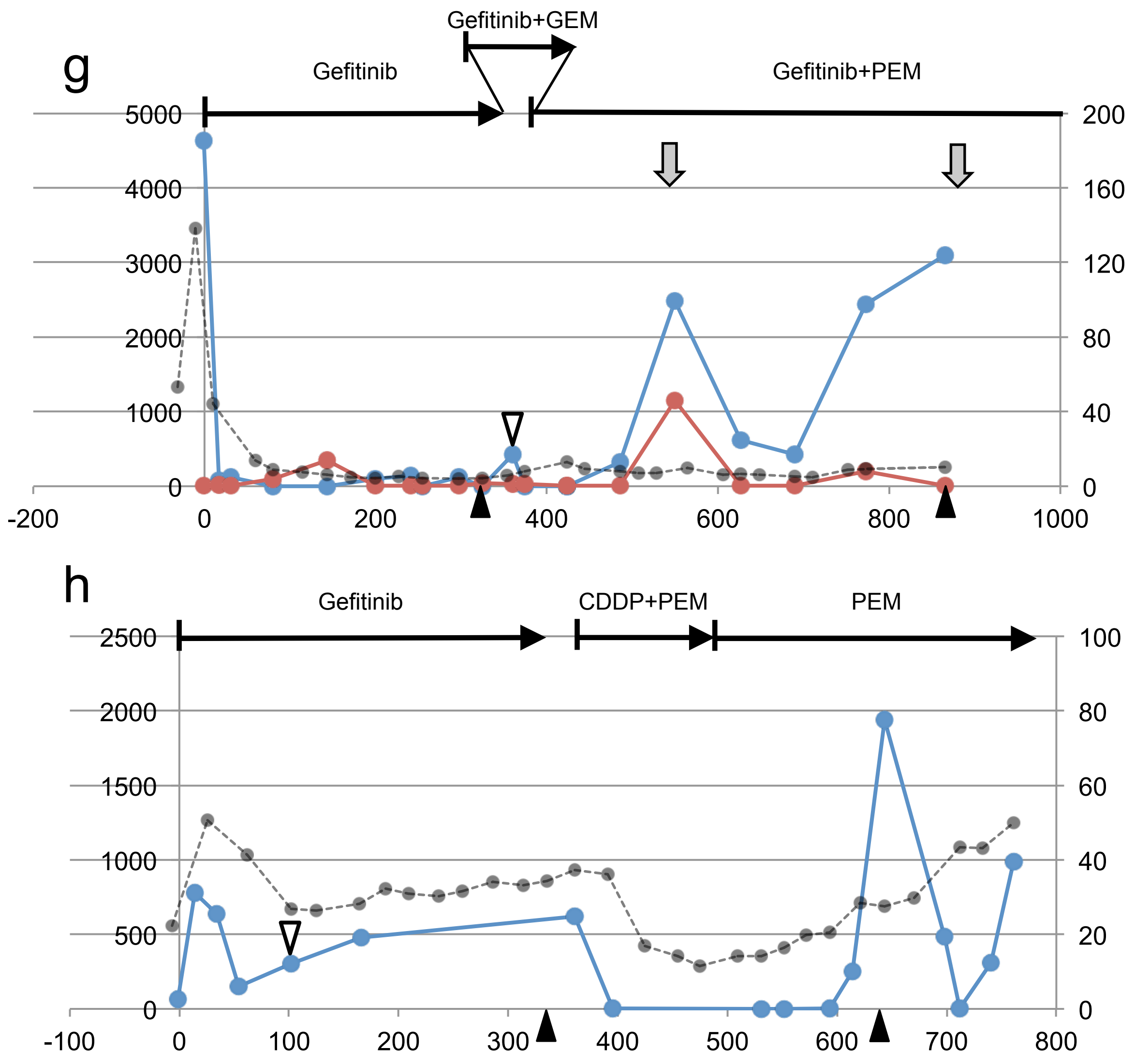

Supplementary Figure S3. ctDNA dynamics of patients not presented in Figures 2 and 3. All patients except patient 15 (type II) belong to type I. The details of panels are the same as those in Figure 2. a, Patient 2. b, Patient 3. c, Patient 4. d, Patient 6. e, Patient 7. f, Patient 8. g, Patient 10. h, Patient 15. The details of panels are the same as those in Figure 2.

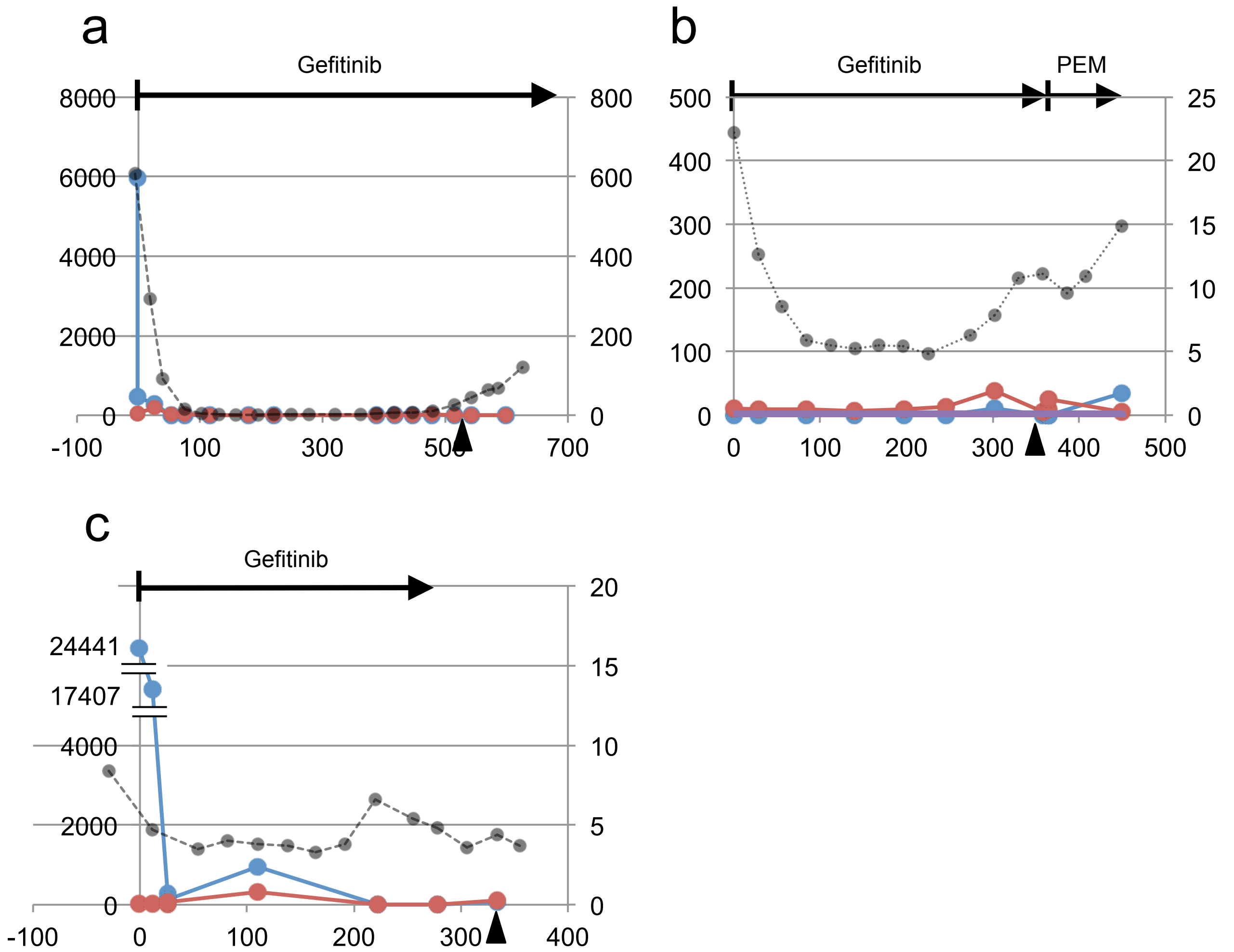

Supplementary Figure S4. Representative examples of ctDNA dynamics of type III patients. The details of panels are the same as those in Figure 2. a, Patient 22. b, Patient 23. c, Patient 28. The details of panels are the same as those in Figure 2.
